# Causal relationship between systemic circulatory inflammatory regulators and intervertebral disc degeneration: A bidirectional 2-sample Mendelian randomization study

**DOI:** 10.1097/MD.0000000000039521

**Published:** 2024-09-06

**Authors:** Zi-Xuan Chen, Bo Xu, Ze-Ling Huang, Yu-Jiang Liu, Yu-Wei Li, Bin-Jie Lu, Jun Lin, Xian-Da Zhang, Xiao-Feng Shen

**Affiliations:** a Department of Orthopedics, Suzhou TCM Hospital Affiliated to Nanjing University of Chinese Medicine, Suzhou, Jiangsu, China; b Nanjing University of Chinese Medicine, Nanjing, Jiangsu, China; c Anhui University of Chinese Medicine, Hefei, Anhui, China.

**Keywords:** genetic relationships, infection susceptibility, inflammatory regulators, intervertebral disc degeneration, Mendelian randomization

## Abstract

In the context of the development of intervertebral disc degeneration (IDD), inflammatory mediators play a pivotal role. Nevertheless, due to the influence of the inflammatory microenvironment, the causal relationship between specific inflammatory mediators and the development of IDD remains uncertain. The understanding of the causal relationship between inflammatory mediators and IDD is of great importance in preventing and delaying disc degeneration in the future. We utilized genetic data concerning systemic circulating inflammatory regulators obtained from a Genome-Wide Association Study (GWAS) analyzing 41 serum cytokines in a cohort of 8293 individuals from Finland. The genetic data for IDD were derived from the most recent GWAS summary statistics conducted within the FinnGen consortium, encompassing 37,636 IDD cases and 270,964 controls. Our analysis employed bidirectional 2-sample Mendelian randomization (MR) techniques, which included several MR methods such as MR Egger, weighted median, inverse variance weighted, weighted mode, and simple mode. Additionally, the MR-PRESSO method was employed to identify horizontal pleiotropy, heterogeneity was quantified using the Cochran *Q* statistic, and MR-Egger intercept analysis was performed to assess pleiotropy. We established causal relationships between 3 specific inflammatory factors and IDD. Elevated levels of MIP-1β (OR = 0.956, 95% CI: −0.08 to −0.006; *P* = .02) and IFN-G (OR = 0.915, 95% CI: −0.16 to −0.02; *P* = .01) expression were associated with a reduced risk of IDD. Conversely, genetic susceptibility to IDD was linked to a decrease in IL-13 levels (OR = 0.967, 95% CI: −0.063 to −0.004; *P* = .03). In this study, we have identified inflammatory factors that exhibit a causal relationship with the onset and progression of IDD, as supported by genetic predictions.

## 1. Introduction

Lower back pain (LBP) stands as the most prevalent musculoskeletal disorder, ranking first in terms of disability.^[1]^ Intervertebral disc degeneration (IDD) constitutes a significant cause of LBP, with approximately 40% of chronic LBP cases being associated with IDD.^[2]^ In recent years, degenerative spinal conditions have triggered severe LBP among middle-aged and even younger individuals, thus evolving into a public health concern. This phenomenon greatly impacts the quality of life and imposes a substantial economic burden on society. The global prevalence of LBP stands at 9.4%, and the associated healthcare costs have surged by 10% over the past 43 years.^[3]^ Nevertheless, the pathogenic mechanisms of IDD remain unclear, and a deeper understanding of its etiology is paramount for the prevention and treatment of LBP.

Inflammatory environments are believed to play a pivotal role in the progression of IDD,^[4]^ yet their mechanisms are complex and not entirely elucidated. In specific circumstances, the inflammatory microenvironment plays a crucial role in maintaining a stable environment within the intervertebral disc (IVD). For instance, there exists a delicate balance between IL-1β and its inhibitor, IL-1Ra, within the normal IVD to sustain the homeostasis of the extracellular matrix (ECM).^[5]^ Simultaneously, IL-1β and TNF-α, at particular concentrations, cooperatively uphold the equilibrium between synthesis and degradation of the extracellular matrix.^[6]^ However, various factors can disrupt the biomechanical structure and cellular homeostasis within the normal IVD, stimulating the upregulation of multiple inflammatory factors, thus giving rise to an inflammatory microenvironment. Furthermore, the IVD’s own inflammatory microenvironment recruits additional exogenous inflammatory cells, further exacerbating the severity of IDD.^[7]^ These pieces of evidence suggest that inflammation serves as both a cause of IDD and a consequence of a vicious cycle following IDD’s onset.

Nonetheless, current research faces various limitations: Replicating the complex inflammatory microenvironment within the IVD presents a significant challenge. It is difficult to eliminate the interplay between individual factors. Observing individual inflammatory factor levels at specific time points in clinical settings may not provide a comprehensive reflection of the overall inflammatory state during the disease process. Hence, there is an immediate need for an accurate assessment of the relationship between circulating inflammatory factors and the risk of IDD.

Mendelian randomization (MR) leverages genetic variations as instrumental variables (IVs) to estimate causal relationships between exposures, intermediates, and outcomes, thereby mitigating biases stemming from residual confounding and reverse causality, which can be encountered by conventional epidemiological approaches.^[8]^ Numerous prior investigations have successfully established causal links between circulating inflammatory factors and various elements.^[9–11]^ Consequently, in this 2-sample MR analysis, conducted bidirectionally, our primary aim is to explore the causal connection between systemic circulatory inflammatory regulators and the development of IDD. Revealing the relationship between the 2 at the genetic level has important implications for future targeted conservative treatment of degenerative discs or accelerated rehabilitation of patients after surgery.

## 2. Materials and methods

### 2.1. Study design

**Figure 1.**
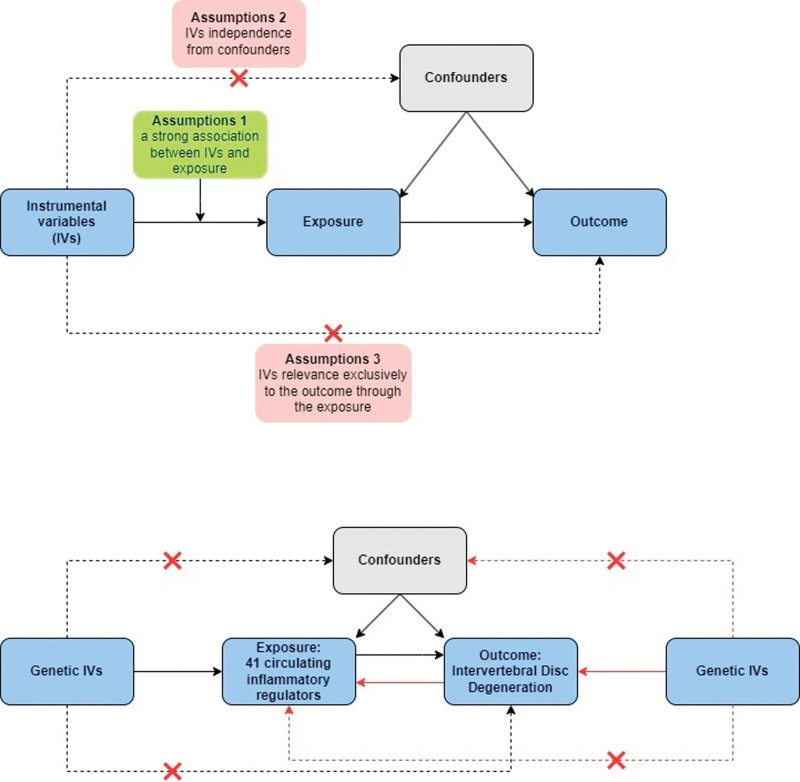
Research design.

The overall workflow of this study is depicted in the figure. MR analysis requires meeting 3 assumptions: a strong association between IVs and exposure, IVs independence from confounders, and IVs relevance exclusively to the outcome through the exposure.^[12]^ This study utilizes bidirectional 2-sample MR analysis to thoroughly examine the causal relationship between systemic inflammatory regulators and IDD. Our research design is summarized in Figure 1.

### 2.2. Data sources

Both datasets employed in this MR analysis are extracted from summary data accessible to the public, originating from Genome-Wide Association Studies (GWAS). We obtained the most recent GWAS summary statistics for IDD from the FinnGen consortium R9 version (r9.finngen.fi), comprising 37,636 cases and 270,964 controls.^[13]^ Data for 41 circulating inflammatory regulators were extracted from GWAS involving 8293 individuals across 3 distinct cohorts.^[14]^

### 2.3. Single nucleotide polymorphism filtering

The core assumptions of MR require that all single nucleotide polymorphisms (SNPs) act as strong and independent predictors of the exposure, demonstrating genome-wide statistical significance. In our study, which examined 41 inflammatory factors and IDD, we chose a relatively lenient yet highly significant *P*-value threshold of 5 × 10^−6^. This decision was made because a more stringent threshold of 5 × 10^−8^ would exclude a substantial number of SNPs, making the study unfeasible.^[15]^

To address linkage disequilibrium, we applied an *R*^2^ < 0.001 and a physical distance of 10,000 kb. Palindrome SNPs were removed from the analysis. Additionally, we considered genetic variants with an *F*-value (calculated as *F* = β²_exposure/SE²_exposure) below 10 as weak IVs and excluded them from the study. These methodological steps strengthened the association between IVs and exposure factors, thereby enhancing the scientific validity and credibility of our MR analyses.

### 2.4. Bidirectional MR analysis

We employed 5 MR methods to calculate the relationship between the impact of SNPs on the outcome and their influence on exposure, consolidating the results obtained for each SNP to thoroughly evaluate the causal connection between exposure and outcome. The 5 MR methods include Inverse Variance Weighted (IVW), MR-Egger regression, weighted median method, simple mode, weighted mode. Among these, the results obtained through the IVW method serve as the primary reference in this study,^[16]^ with *P* < .05 considered statistically significant evidence. We detected horizontal pleiotropy through a global test using the MR-PRESSO method and corrected potential pleiotropic outliers by removing SNP outliers when necessary.^[17]^ Finally, we quantified heterogeneity through the Cochran *Q* statistic and conducted MR-Egger intercept analysis to test for pleiotropy, with *P* > .05 serving as a reference value indicating no significant heterogeneity and pleiotropy.^[18,19]^

MR analysis, including 2-sample and multivariable MR, was performed using the TwoSampleMR package in R software (version 4.2.2).

## 3. Results

### 3.1. Causal effects of 41 circulating inflammatory regulators on IDD

**Figure 2.**
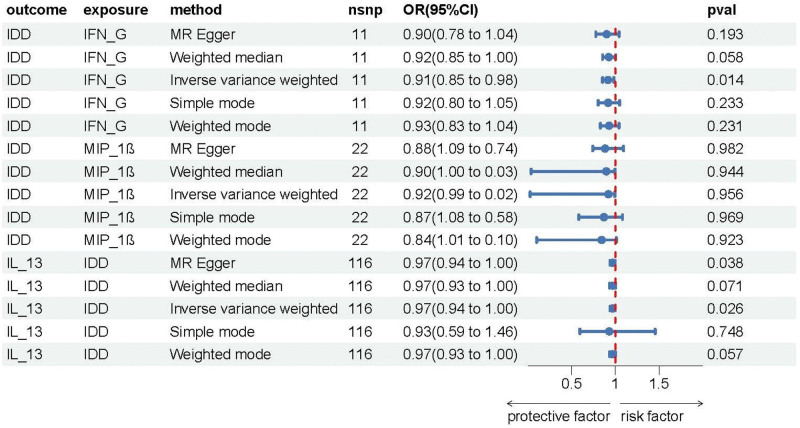
Effect of different exposures on outcomes.

**Figure 3.**
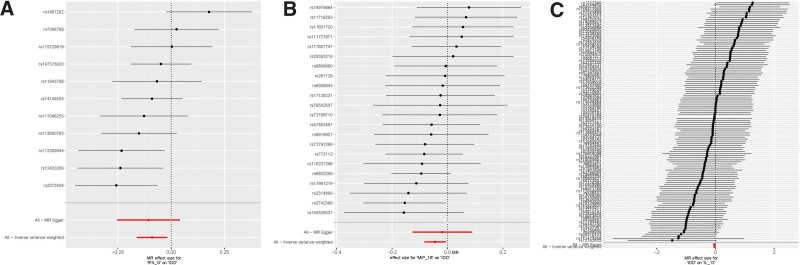
Forest plot of the association between systemic circulating inflammatory modulators and IDD. (A) IFN-G and IDD; (B) MIP-1β and IDD; (C) IDD and IL-13.

**Figure 4.**
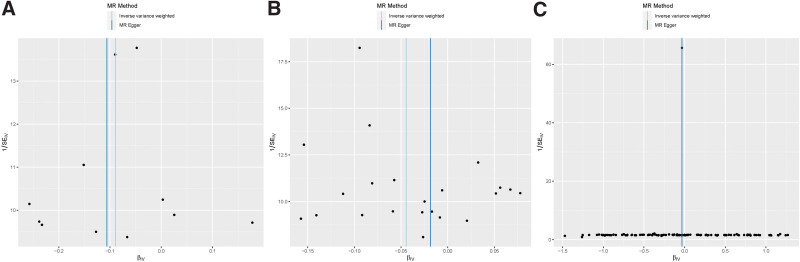
Funnel plots of the association between systemic circulating inflammatory regulators and IDD. (A) IFN-G versus IDD; (B) MIP-1β versus IDD; (C) IDD versus IL-13.

**Figure 5.**
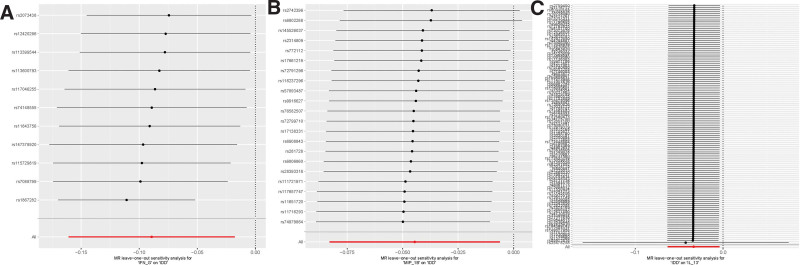
Leave-one-out sensitivity analysis of the relationship between systemic circulating inflammatory modulators and IDD. (A) IFN-G and IDD; (B) MIP-1β and IDD; (C) IDD and IL-13.

**Figure 6.**
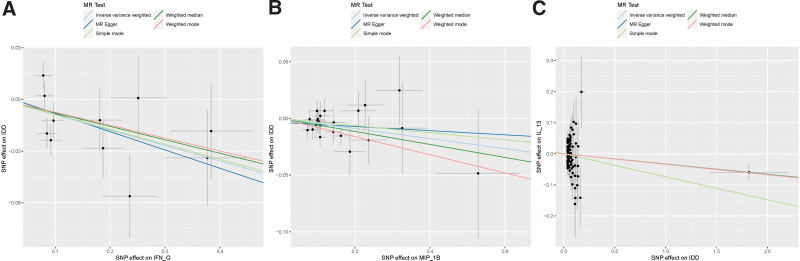
Scatter plots of the association between systemic circulating inflammatory modulators and IDD. (A) IFN-G versus IDD; (B) MIP-1β versus IDD; (C) IDD versus IL-13.

Out of the initial 41 systemic circulatory inflammatory regulators, a total of 428 SNPs were selected. The primary prediction method, IVW MR, demonstrated that increased expression levels of 2 inflammatory regulators, MIP-1β (OR = 0.956, CI: −0.08 to −0.006; *P* = .02) and IFN-G (OR = 0.915, CI: −0.16 to −0.02; *P* = .01), were associated with a decreased risk of IDD. Although not exhibiting statistical significance, the remaining 4 MR analysis methods provided some level of support for the aforementioned results (see Table S1, Supplemental Digital Content, http://links.lww.com/MD/N529; Figure 2 to 6). The Cochran *Q* method did not detect heterogeneity (*P* > .05), and both MR-Egger and MR-PRESSO tests did not reveal horizontal pleiotropy (*P* > .05). The “leave-one-out” method involves systematically removing each SNP 1 at a time, calculating the combined effect of the remaining SNPs, and observing whether the results vary after each SNP is excluded. If significant changes in the results occur when a particular SNP is removed, it suggests that this specific SNP has a substantial impact on the overall findings. To ensure that the results are not unduly influenced by any single SNP, we conducted a “leave-one-out” sensitivity test. This analysis revealed that the overall results remained largely consistent after excluding each SNP, demonstrating the robustness and reliability of our findings.

### 3.2. Causal effects of IDD on 41 circulating inflammatory regulators

To assess the reverse causal effects, we extracted 135 SNPs associated with IDD, with significance levels at *P* < 5 × 10^−6^. As some SNPs were not applicable to certain cytokines, varying numbers of SNPs were used for different cytokines, which can be found in Table S2, Supplemental Digital Content, http://links.lww.com/MD/N529. Using the IVW method, We observed that an increased genetic predisposition to IDD correlated with a reduction in the levels of 2 factors: GROA (OR = 0.961, 95% CI: −0.071 to −0.008; *P* = .02) and IL-13 (OR = 0.967, 95% CI: −0.063 to −0.004; *P* = .03) (Figs. 2-6). As the analysis using the simple mode in GROA yielded results contrary to the other 4 methods, we decided to exclude GROA. Sensitivity analysis was also performed, which did not detect heterogeneity or horizontal pleiotropy (*P* > .05), and the leave-one-out sensitivity test did not reveal variation in the overall outcome.

## 4. Discussion

This study employed a 2-sample MR analysis, utilizing data from the FinnGen project and GWAS data, to assess the causal association between 41 circulating inflammatory regulators and IDD. The study results indicated that higher levels of MIP-1β and IFN-γ were associated with a lower risk of IDD, while the reverse causal analysis revealed an association between genetic susceptibility to IDD and decreased levels of IL-13.

Inflammation consistently plays a significant role in the initiation and progression of IDD.^[20]^ Various factors, both intrinsic and extrinsic to the intervertebral disc, can create an inflammatory microenvironment. These factors include microcrystals, degradation products of the ECM, mechanical loading, and immune cell infiltration. They stimulate disc cells to produce various inflammatory mediators, such as cytokines, chemokines, NLRP3 inflammasomes, prostaglandin E2 (PGE2), IFN-γ.^[21]^ These inflammatory mediators contribute to the pathology of IDD in multiple ways. They promote the expression and activity of ECM-degrading enzymes, leading to structural and functional damage to the ECM. ^[22]^ Additionally, they induce senescence, apoptosis, and abnormal proliferation of disc cells^[7]^; Inflammatory mediators also increase neurovascular generation and the production of pain-related factors, which cause nerve fiber growth and innervation within the intervertebral disc.^[23]^ Therefore, regulating the inflammatory microenvironment and restoring the balance of cytokines are effective strategies for controlling IDD.

Previous observational studies have indicated that the expression of inflammatory factors such as IL-1β and TNF-α significantly increases in IDD, and their expression levels are positively correlated with patient age and the severity of IDD.^[24–26]^ Furthermore, a higher expression of the NLRP3 inflammasome in IDD can mediate the production of various inflammatory cytokines, further promoting IDD development.^[25]^

However, these observational studies might be subject to confounding factors or reverse causality that could distort the true causal relationship. For example, Weber et al^[27]^ found that serum levels of IL-6 in IDD patients showed no significant association with the severity of IDD as indicated by MRI. These findings emphasize the complex relationship between inflammation and IDD.

MIP-1β, also known as CCL4, is a CC chemokine that plays a role in inflammation and host defense mechanisms by interacting with its specific receptor CCR5.^[28]^ Previous studies have suggested that MIP-1β expression levels are significantly elevated in severely degenerated NP tissue compared to mildly degenerated NP tissue.^[29,30]^ IFN-γ, also known as IFN-gamma, is 1 of the main inducers of M0 macrophage polarization.^[31]^ Gorth et al^[32]^ found that transgenic mice with overexpressed TNF-α exhibited early spontaneous IVD protrusion and elevated levels of IL-6, TNF-α, and IFN-γ upon NP exposure. Furthermore, research has shown that IFN-γ can increase the expression of TNF-α, IL-1β, COX-2, MMP-3, MMP-13, ADAMT-4, and ADAMT-5 in IVD cells.^[33]^ Interleukin-13 (IL-13) is an anti-inflammatory factor produced by Th2 cells, macrophages, and mast cells, and it participates in tissue remodeling and fibrosis through activation of the JAK/STAT signaling pathway.^[34]^ IL-13 receptors are expressed on macrophages and can inhibit M1 polarization while promoting M2 polarization, serving anti-inflammatory and tissue repair functions.^[35]^ Currently, there is limited research on the relationship between IL-13 and IDD.

In contrast to many previous observational studies, our study suggests that higher levels of IFN-γ and MIP-1β may lead to a decreased genetic risk for IDD, which could be attributed to the immunoregulation and tissue repair abilities of inflammatory factors on degenerated intervertebral discs. Yu et al^[36]^ proposed that the loss of immune privilege caused by intervertebral disc lesions could be a significant factor in the development of IDD and sciatica. While the infiltration of inflammatory cells, growth factors, and cytokines may promote IDD, the functions of specific immune cells may aid in the regeneration of damaged disc tissues and the restoration of immune privilege. It has been reported that IFN-γ-induced inflammation of mesenchymal stem cells may improve their ability to suppress T-cell proliferation, serving an anti-inflammatory and tissue repair function.^[37]^ In the tumor microenvironment, IFN-γ and MIP-1β have shown dual roles in both antitumor and pro-tumor effects.^[38,39]^ Whether these roles are relevant to the process of IDD remains an avenue for further research. In the assessment of reverse causality, we found that the progression of IDD is associated with decreased levels of IL-13. We speculate that during the IDD process, the destabilization of the intracellular environment leads to increased expression of pro-inflammatory factors such as TNF-α and IL-1β, while concurrently resulting in a decrease in the expression of anti-inflammatory factors such as IL-13, ultimately creating an inflammatory microenvironment within the IVD. Therefore, activating the expression of anti-inflammatory factors like IL-13 within the IVD may be a strategy to slow the progression of IDD.

Establishing a causal relationship between inflammatory factors and IDD holds significant potential for advancing targeted therapies and preventive strategies. Understanding these relationships enables the development of precision medicine approaches, including the use of biologic therapies such as monoclonal antibodies and gene therapy to modulate specific inflammatory mediators like IL-1β, TNF-α, and IFN-γ. These targeted therapies promise improved efficacy and reduced side effects compared to nonspecific anti-inflammatory treatments. Additionally, existing drugs that target these inflammatory pathways can be repurposed, accelerating the availability of new treatments. Early identification of genetic markers for heightened inflammatory responses facilitates risk stratification and early intervention, crucial for preventing significant degeneration. This allows for personalized preventive measures, including lifestyle modifications and early pharmacologic interventions tailored to high-risk individuals. Public health strategies can also be informed by these insights, guiding recommendations for physical activity, occupational health, and nutrition to reduce the incidence of IDD. Research and clinical applications benefit from an enhanced understanding of IDD pathophysiology. This understanding leads to better-designed clinical trials that utilize identified biomarkers as endpoints, improving their accuracy and relevance. Moreover, a holistic treatment approach that integrates anti-inflammatory strategies with physical therapy, surgical interventions, and other treatments can provide more comprehensive care plans for patients. Overall, recognizing the causal role of inflammation in IDD opens new avenues for innovative therapies and preventive measures. This understanding not only advances the development of targeted and effective medical interventions but also supports the creation of personalized and population-based strategies to manage and prevent IDD, ultimately improving patient outcomes and quality of life.

This study represents the first application of MR to examine the link between inflammation and IDD. This method helps overcome the problems of reverse causality bias that are common in traditional observational studies. Furthermore, we imposed strict study restrictions to reduce heterogeneity and pleiotropy in the outcome measures, providing reliable causal relationship estimates. Another notable aspect is the wide availability of serum samples in clinical studies, making our findings amenable to further validation and application. While the typical genome-wide significance *P*-value threshold is set at 5 × 10^−8^, our study used a relatively lenient threshold of 5 × 10^−6^ to explore more possibilities.

MR is a robust method for inferring causality, yet it is imperative to recognize its inherent limitations. MR relies on key assumptions: genetic variants must exhibit a strong association with the exposure (relevance), SNPs should be independent of confounders influencing both the exposure and the outcome (independence), and genetic variants should impact the outcome solely through the exposure, not via alternative pathways (exclusion restriction). Violations of these assumptions can result in biased estimates due to weak instruments, residual confounding, and pleiotropy, wherein a single genetic variant affects multiple traits. Despite genetic randomization, unmeasured confounding persists as a concern, as pleiotropy and population stratification – variations in allele frequencies across subpopulations – can introduce bias if not properly controlled. Furthermore, measurement error in the exposure can cause attenuation bias, underestimating the true effect of the exposure on the outcome, underscoring the necessity of accurate exposure measurement. The generalizability of MR study results is also limited, given the potential influence of gene-environment interactions or population-specific allele frequencies, thus necessitating caution when extrapolating findings to diverse demographic groups. Lastly, large sample sizes are often essential to detect small effects, particularly when genetic variants account for a minor proportion of the variance in the exposure; inadequate sample sizes may result in insufficient power and inconclusive outcomes.

## 5. Conclusion

In conclusion, this 2-sample MR study suggests a causal relationship between inflammation and IDD. Higher levels of MIP-1β and IFN-γ appear to reduce the risk of IDD, and the progression of IDD is associated with a decrease in IL-13 levels. These findings provide new insights into strategies for preventing and delaying IDD progression.

## Author contributions

**Funding acquisition:** Xiao-Feng Shen.

**Supervision:** Xiao-Feng Shen.

**Writing – original draft:** Zixuan Chen.

**Writing – review & editing:** Bo Xu, Ze-Ling Huang, Yu-Jiang Liu, Yu-Wei Li, Bin-Jie Lu, Jun Lin, Xian-Da Zhang, Xiao-Feng Shen.

## Supplementary Material


